# Commentary to “Polylactic Acid: Is It Everything the Same? [sic]”

**DOI:** 10.1111/jocd.16603

**Published:** 2024-09-26

**Authors:** Jose Eduardo Chicarelli Martin

**Affiliations:** ^1^ Medical Affairs Department Rennova São Paulo Brazil

**Keywords:** biostimulator, collagen, poly‐L‐lactic acid, safety

Dear Editor,

We would like to express our sincere appreciation for the efforts that went into your recent manuscript “Polylactic acid: Is it everything the same? [sic]” [1]. The increasing use of poly‐L‐lactic acid (PLLA) as a treatment option has indeed gained significant attention in both clinical practice and research. As more patients seek PLLA‐based treatments, the importance of ensuring patient safety and achieving optimal outcomes cannot be overstated. Your work contributes to this growing body of literature by highlighting the need for a deeper understanding of the differences between available PLLA products.

While we acknowledge the potential value of your in vitro analyses, which were undoubtedly carried out with the best of intentions, we feel compelled to highlight several concerns regarding the methodology and conclusions of your study. It is in this spirit of constructive scientific discourse that we allow ourselves to respectfully offer the following remarks:

- While in vitro studies serve as a fundamental foundation in scientific research, the conditions under which your analysis was conducted do not appear to be entirely representative of clinical realities. Specifically, your study analyzed the PLLA particles only 1 day after dilution, which differs from the typical two‐dimensional (2D) or three‐dimensional cell culture condition and fails to account for the cellular activity and physiological interactions in vivo that are of essential importance in this specific type of injection‐based aesthetic treatment [2]. In general, the behavior of biomaterials in vitro does not always correlate directly with their performance in vivo, particularly when it comes to particle degradation, tissue interaction, and overall biocompatibility. The physical structure of PLLA micro particles is complex, with different microstructures—namely, solid, double‐walled, hollow, and porous microspheres—imparting distinct properties that influence their suitability as injectable carriers [3]. For a (more) meaningful in vitro evaluation, it would have been advisable to monitor not only the morphology of the PLLA particles but also other critical factors such as pH changes in the medium, molecular weight variations, thermodynamic properties, and the chemical structure over time. Such parameters are essential for predicting PLLA's degradation profile and its potential effects on the surrounding tissue. Without these additional insights, any conclusions drawn from a very short‐term, one‐dimensional analysis risk being incomplete and/or misleading.
- In addition to the methodological concerns, we feel obliged to also address the way in which the available literature is presented, particularly regarding the complication rates associated with Elleva and Sculptra. On the one hand, it is important to note that Elleva has been used successfully in previous studies, yielding positive outcomes with minimal complications when used appropriately [4, 5]. On the other hand, the selective reporting of data from a single study, which itself has a series of limitations, does not provide a fair representation of the current body of evidence [6]. First, the cited study lacked standardized follow‐up protocols and apparently offered no mid‐ or long‐term data. Second, the study failed to provide adequate information regarding the exact product application, including details on the volume injected, the number of sessions, or the specific injection techniques used. Third, no data on post‐treatment care were provided. Fourth, there was no information on the health status of patients prior to treatment, whether they had undergone previous treatments, if they received additional treatments post‐injection, or on the injector's experience level, all of which could significantly influence the reported outcomes. Fifth, the study did not capture the core of aesthetic medicine, that is, patient‐reported outcomes. Sixth, arguably the most glaring limitation was the lack of clarity regarding the usage frequency of each product examined. The cited study reported only the number of complications and the proportion of complications associated with each product but did not document how often each product was actually used. This omission introduces a significant risk of misrepresentation and disproportionately skewed data. The validity of the study is further called into question when considering the broader body of literature on PLLA and its associated risks. In fact, other studies documented the (significant) risk of nodule formation following the use of Sculptra [7, 8]. The portrayal of Elleva as having a markedly higher risk of complications may, therefore, be an oversimplification that does not accurately reflect the full scope of available evidence. We would like to seize this opportunity and stress the importance of conducting a thorough and systematic review of all existing literature to provide a well‐rounded and balanced comparison of product characteristics and outcomes. It is essential that conclusions are drawn from a comprehensive synthesis of the complete body of evidence, thus minimizing any potential biases from selective data inclusion. Such an approach not only upholds scientific integrity but also ensures that statements made are impartial and rooted in the best available evidence.
- It is unclear whether the authors performed their own ultrasound comparison of Elleva and Sculptra or if they merely reference a study that reported the acoustic shadowing. If so, it is important to highlight that this cited study used freshly thawed cadavers for echographic evaluation, which—albeit useful for initial assessments—fails to fully replicate the metabolic activity and conditions in a living organism. In addition, the cited study did not conclusively explain the cause of the acoustic shadow; instead, the authors actually speculated that Elleva's slightly higher carboxymethylcellulose content (0.63 vs. 0.60 in Sculptra) might be responsible [9]. Of note, Sigrist's group reiterated this theory in one of their subsequent studies, where they, strikingly, also noted a more homogeneous distribution of PLLA particles with Elleva compared to Sculptra. According to the authors, this may facilitate a more even fibroplasia, which will drive a “more effective neocollagenesis response […] and a possible better therapeutic result” [10].
- We would also like to highlight the significant advances Elleva brought to the market. As a more recent entrant, Elleva represents innovation in the field of biostimulatory products, offering practitioners and patients an alternative with distinct product characteristics and properties. Innovation inherently brings about differences, which should—we definitely agree on this point—be examined objectively and scientifically, without any prejudice. The potential of Elleva to expand treatment options and tailor approaches to individual patient needs and preferences is a noteworthy aspect that we believe was underrepresented in your article.

In conclusion, while we commend your efforts to contribute to the understanding of PLLA products, we must respectfully assert that your study, though well‐intentioned, in our opinion does not fully achieve its goal of providing a balanced and scientifically robust comparison between Elleva and Sculptra. We hope that our constructive and considerate critique will be seen as an opportunity to hone the discussion surrounding these products and encourage further research that prioritizes patient safety and satisfaction.

We appreciate your attention to these concerns and look forward to continued dialog on this important topic.

## Author Contributions

J.E.C.M.: conceptualization and writing – original draft.

## Disclosure

J.E.C.M. is a dermatologist with a private practice and director of the Medical Affairs Department at Rennova.
